# Role of circadian gene Clock during differentiation of mouse pluripotent stem cells

**DOI:** 10.1007/s13238-016-0319-9

**Published:** 2016-09-23

**Authors:** Chao Lu, Yang Yang, Ran Zhao, Bingxuan Hua, Chen Xu, Zuoqin Yan, Ning Sun, Ruizhe Qian

**Affiliations:** 1 Department of Physiology and Pathophysiology, School of Basic Medical Sciences, Fudan University, Shanghai, 200032 China; 2 Research Center on Aging and Medicine, Fudan University, Shanghai, 200032 China; 3 State Key Laboratory of Medical Neurobiology, Fudan University, Shanghai, 200032 China; 4 Department of Orthopedics, Zhongshan Hospital, Fudan University, Shanghai, 200032 China

**Keywords:** Circadian gene *Clock*, mouse embryonic stem cells, gene knockout, pluripotency, cell proliferation, cell apoptosis, cell differentiation

## Abstract

Biological rhythms controlled by the circadian clock are absent in embryonic stem cells (ESCs). However, they start to develop during the differentiation of pluripotent ESCs to downstream cells. Conversely, biological rhythms in adult somatic cells disappear when they are reprogrammed into induced pluripotent stem cells (iPSCs). These studies indicated that the development of biological rhythms in ESCs might be closely associated with the maintenance and differentiation of ESCs. The core circadian gene *Clock* is essential for regulation of biological rhythms. Its role in the development of biological rhythms of ESCs is totally unknown. Here, we used CRISPR/CAS9-mediated genetic editing techniques, to completely knock out the *Clock* expression in mouse ESCs. By AP, teratoma formation, quantitative real-time PCR and Immunofluorescent staining, we did not find any difference between *Clock* knockout mESCs and wild type mESCs in morphology and pluripotent capability under the pluripotent state. In brief, these data indicated *Clock* did not influence the maintaining of pluripotent state. However, they exhibited decreased proliferation and increased apoptosis. Furthermore, the biological rhythms failed to develop in *Clock* knockout mESCs after spontaneous differentiation, which indicated that there was no compensational factor in most peripheral tissues as described in mice models before (DeBruyne et al., 2007b). After spontaneous differentiation, loss of CLOCK protein due to *Clock* gene silencing induced spontaneous differentiation of mESCs, indicating an exit from the pluripotent state, or its differentiating ability. Our findings indicate that the core circadian gene *Clock* may be essential during normal mESCs differentiation by regulating mESCs proliferation, apoptosis and activity.

**Electronic supplementary material:**

The online version of this article (doi:10.1007/s13238-016-0319-9) contains supplementary material, which is available to authorized users.

## INTRODUCTION

Circadian rhythm is a fundamental biological system in humans, animals and plants that regulates various physiological functions such as sleep–wake cycle, energy metabolism, cell division, post-transcriptional regulation (Koike et al., 2012; Cardinal-Aucoin and Steel, 2016; Lopez-Minguez et al., 2016; McAlpine and Swirski, 2016; Nitschke et al., 2016), and endocrine system (Gachon et al., 2004; Lowrey and Takahashi, 2004). The master circadian clock, located in the suprachiasmatic nucleus (SCN), is an essential activator of downstream molecular events critical to the generation of circadian rhythms (Dunlap, 1999). The master clock is regulated by four major groups of genes: *Clock, Bmal1, Cry* (*Cryptochrome* 1 and 2), and *Per* (*Period* 1 and 2). These core circadian genes are regulated in a transcription-translation feedback loop (TTFL) (Xue et al., 2016).

The core circadian genes regulate critical aspects of cellular processes in many organs. Mutations in the circadian genes affect circadian activities and are hazardous to health (King et al., 1997; Kovanen et al., 2010). For instance, some recent studies have shown associations between clock genes and chronic inflammation, blood pressure and energy intake in humans, supporting the importance of the circadian rhythm in cardiac physiology (Dashti et al., 2016; Johnston and Ordovas, 2016). Mutations in *Per* genes increase cancer incidence (Fu et al., 2002; Wood et al., 2008; Borgs et al., 2009) and cancer cell proliferation (Borgs et al., 2009). Circadian clock dysfunction has been linked to oxidative stress and age-related neurodegeneration *in vivo* (Witting et al., 1990; Hu et al., 2009; Wyse and Coogan, 2010). Adult stem cell functions are also regulated by circadian oscillations (Casanova-Acebes et al., 2013; Janich et al., 2013; Karpowicz et al., 2013).

Circadian rhythms in the suprachiasmatic nucleus (SCN) manifest only a few days before birth (Yagita et al., 2010). Bmal1 is expressed in mice during embryogenesis without oscillation (Johnson et al., 2002). It may have other key roles instead of circadian rhythm control during embryogenesis (Yang et al., 2016). Indeed, circadian rhythms do not occur in embryonic stem cells (ESCs) but develop when ESCs exit from the pluripotent state and differentiate to downstream cells (Yagita et al., 2010). Conversely, biological rhythms in adult somatic cells disappear when they are reprogrammed into induced pluripotent stem cells (iPSCs). To better understand the molecular basis underlying circadian rhythms and ESCs pluripotency and differentiation, we studied the role of the core circadian gene *Clock* in the maintenance, differentiation and development of circadian rhythms in mouse embryonic stem cells (mESCs). Using the CRISPR/CAS9 genome editing tool, we completely knocked out the *Clock* gene in mESCs. We found that *Clock* knockout mESCs still exhibited normal clonal morphology and pluripotency, which indicated that *Clock* gene might not be required for regular maintenance of mESCs. However, *Clock* knockout significantly decreased the proliferation and increased the apoptosis of mESCs. Furthermore, the biological rhythms failed to develop in *Clock* knockout mESCs after spontaneous differentiation. Loss of CLOCK protein due to *Clock* gene silencing in mESCs triggered their spontaneous differentiation, leading to stronger expression of genes in the three embryonic germ layers downstream.

Our findings suggest that the core circadian gene *Clock* influences mESCs differentiation process by regulating mESCs proliferation, apoptosis and activity.

## RESULTS

### Knock out of *Clock* in mESCs using CRISPR/CAS9 genomic editing

**Figure 1:**
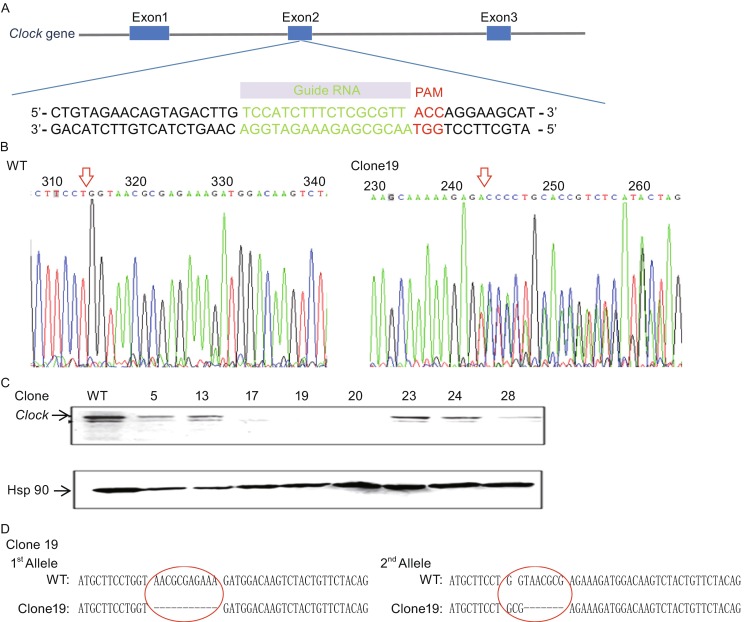
**Generation of** ***Clock*** **knockout mESC line using CRISPR/CAS9 gene editing**. (A) Schematic diagram of the 5′ end of the *Clock* gene (blue bars, exons), the CRISPR/CAS9 gRNA sequence (red bar, the green shaded part), PAM domain (the red shaded part). (B) Representative sequencing showing modifications at the CRISPR/CAS9 cutting site on exon 2 of the mouse *Clock* gene. (C) Western blot revealed CLOCK expression in the positive clones following editing by the CRISPR/CAS9 system. (D) TA cloning results involving a representative homozygous clone (clone 19) show that both alleles of the mouse *Clock* gene were modified on exon 2 by the CRISPR/Cas9 system

To investigate the role of the core circadian gene *Clock* in the maintenance and differentiation of mammalian pluripotent stem cells, we established the *Clock* knockout mESC line using the CRISPR/CAS9 genomic editing tool. This tool enables the efficient construction of knockout alleles via induction of frameshift mutations. In this study, we designed a single gRNA specifically targeting exon 2 in the mouse *Clock* gene (Fig. 1A) and subcloned it into the PX330 CRISPR/CAS9 vectors. Single wild type mESCs were transfected with the subcloned CRISPR/CAS9 plasmids via electroporation. Single colonies with successful genome editing were selected by puromycin and Sanger’s sequencing at the cutting site on exon 2 of the *Clock* gene (Fig. 1B and Fig. S1). We deleted 31 single mESC clones, of which 8 clones (clone 5, clone 13, clone 17, clone 19, clone 20, clone 23, clone 24, and clone 28) showed sequence modifications at the CRISPR/CAS9 cutting site (Table S2). To verify whether the *Clock* gene was completely knocked out, we investigated CLOCK protein expression in the 8 positive clones using Western blot. The results indicated that CLOCK protein expression was totally ablated in 3 clones (clone 17, clone 19, and clone 20) (Fig. 1C). To further confirm whether both alleles of the *Clock* gene were modified in clone 17, clone 19, and clone 20, we performed TA cloning of the PCR products flanking the CRISPR/CAS9 cutting site. The results showed that both alleles were edited at exon 2 of the *Clock* gene in all the 3 homozygous mESC clones (Fig. 1D and Fig. S1). Among the 3 clones, clone 19 was used for further study.

Further, to consider the potential influence of off-target gene modifications (Yee, 2016), we detected the most probable ten off-target modification sites which were designed at the CRISPR website (http://crispr.mit.edu/) (Table S3) and found that there was no off-target gene modification (Fig. S2), which excluded the potential influence of off-target gene modifications.

### *Clock* knockout in mESCs did not affect pluripotency

**Figure 2:**
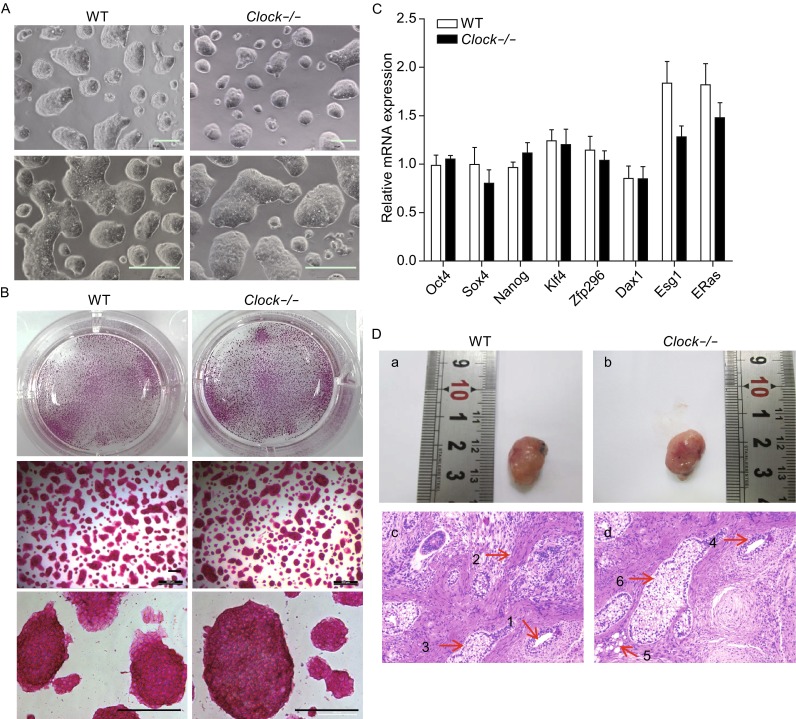
**Knockout of** ***Clock*** **did not affect pluripotency of mESCs**. (A) Phase images of wild type and *Clock*-/- mESCs exhibited compact and round clonal morphology. Bars, 200 µm. (B) Both wild type and *Clock*-/- mESCs stained strongly for alkaline phosphatase. The upper two images represent macroscopic views of a 12-well plate system. Bars, 400 µm. (C) Relative mRNA expression of pluripotent markers *Oct4, Sox2, Nanog, Klf4, Zfp296, Dax1, Esg1,* and *Eras* in wild type and *Clock*-/- mESCs. The mRNA expression levels were normalized to that of the endogenous *GAPDH*. Data represent an average of three independent experiments and are expressed as means ± S.D. Statistical significance was calculated using the Student’s *t*-test: * denotes *P* < 0.05, ** denotes *P* < 0.01, *** denotes *P* < 0.001 compared with the Ctrl group, ns, not significant. No statistical significance was found in the expression levels of the above genes between the wild type and *Clock*-/- mESCs (*P* > 0.05). (D) After respectively injecting 10^7^ wild type mESCs and *Clock* knockout mESCs into immunodeficiency mice, we could observe teratoma of similar size (1–2 cm) formed after four weeks. Six weeks later, Immunohistochemistry manifested that both wild type mESCs and *Clock* knockout mESCs could differentiate to downstream endoderm, mesoderm, and ectoderm layers cells. Among them, arrow 1 and 4 denote gland cells, which belong to endoderm layer cells; arrow 2 denotes smooth muscle cells, and arrow 5 denotes fat vesicle cells, which belong to mesoderm layer cells; arrow 3 and 6 denote sebaceous gland cells, which belong to ectoderm layer cells

**Figure 3:**
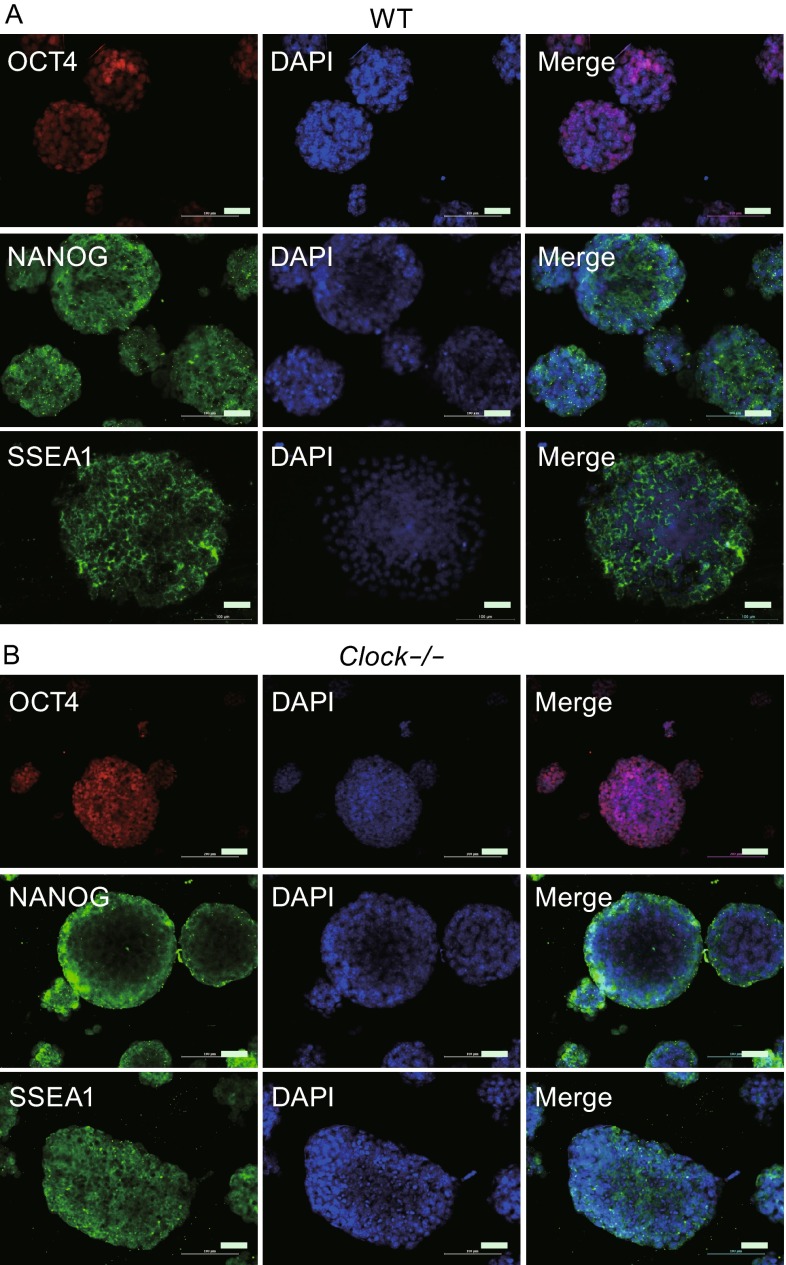
***Clock*** **-/- mESCs express pluripotent markers OCT4, NANOG, and SSEA1**. (A) Immunofluorescence staining of the pluripotent markers OCT4, NANOG, and SSEA1 in wild type mESCs. (B) Immunofluorescence staining of the pluripotency marker OCT4, NANOG, and SSEA1 in *Clock*-/- mESCs. Bars, 100 µm

The *Clock* knockout mESCs exhibited compact clonal morphology without spontaneous differentiation under routine mESC maintenance culture conditions, which was similar to the wild type mESCs (Fig. 2A). To further examine whether silencing of *Clock* gene affected the pluripotency of mESCs, we performed staining for alkaline phosphatase and immunofluorescence of pluripotent markers OCT4, NANOG, and SSEA1. The *Clock* knockout mESCs expressed strong alkaline phosphatase activity as well as OCT4, NANOG, and SSEA1 (Fig. 2B and Fig. 3). Real-time PCR of the mRNA expression levels of pluripotent markers *Oct4, Sox2, Nanog, Klf4, Zfp296, Dax1, Esg1,* and *Eras* revealed no significant differences (*P* > 0.05) from those of wild type mESCs (Fig. 2C). Furthermore, we used teratoma formation assay as a tool for monitoring pluripotency in stem cell research to study the pluripotency of wild type mESCs and *Clock* knockout mESCs *in vivo* (Fig. 2D) (Nelakanti et al., 2015). As shown in Fig. 2D, after injecting 1 × 10^7^ wild type mESCs or *Clock* knockout mESCs into immunodeficiency mice, we could observe teratoma of 1-2 cm formed after four weeks. Six weeks later, we detected the differential capacity of wild type mESCs and *Clock* knockout mESCs by hematoxylin-eosin staining (HE staining). We found that that both wild type mESCs and *Clock* knockout mESCs could differentiate to endoderm, mesoderm, and ectoderm layers cells, which indicated that wild type mESCs and *Clock* knockout mESCs have similar potential of multilineage differentiation *in vivo*. According to these results we got, silencing of *Clock* gene in mESCs didn’t affect their pluripotent nature.

### *Clock* silencing in mESCs decreased cell proliferation rate

**Figure 4:**
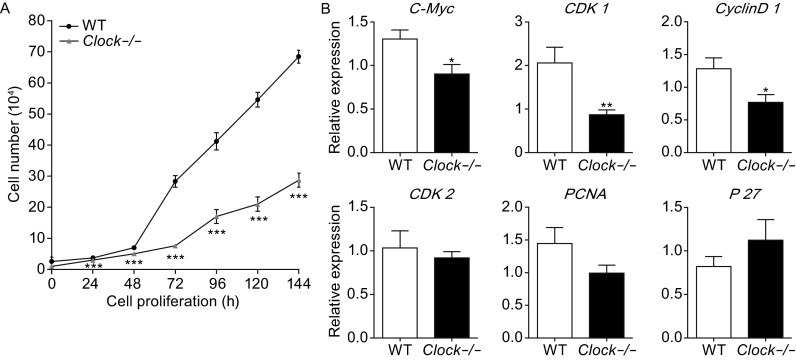
***Clock*** **silencing inhibited proliferation of mESCs**. (A) Cell proliferation curve of the wild type and *Clock*-/- mESCs. *Clock*-/- mESCs exhibited significantly lower proliferation rate than that of the wild type mESCs. (B) Relative mRNA expression levels of cell cycle-related markers *C-Myc, CyclinD1, CDK1, CDK2, PCNA,* and *P27* in wild type and Clock-/- mESCs. The mRNA expression levels were normalized to that of the endogenous *GAPDH*. Data represent an average of three independent experiments and are expressed as means ± S.D. Statistical significance was calculated using the Student’s *t*-test: * denotes *P* < 0.05, ** denotes *P* < 0.01, *** denotes *P* < 0.001 compared with the Ctrl group, ns, not significant

During routine culture, we observed that the proliferation rate of *Clock* knockout mESCs was much slower than that of the wild type mESCs. To further confirm our observation, we performed cell proliferation assays of the *Clock* knockout mESCs and wild type mESCs at different time points. As shown in Fig. 4A, the *Clock* knockout slowed the proliferation rate of mESCs significantly compared with that of the wild type mESCs (*P* < 0.001). These results indicated that *Clock* knockout negatively affected the proliferation of mESCs.

### *Clock* knockout in mESCs slowed down the cell cycle and enhanced cell death

**Figure 5:**
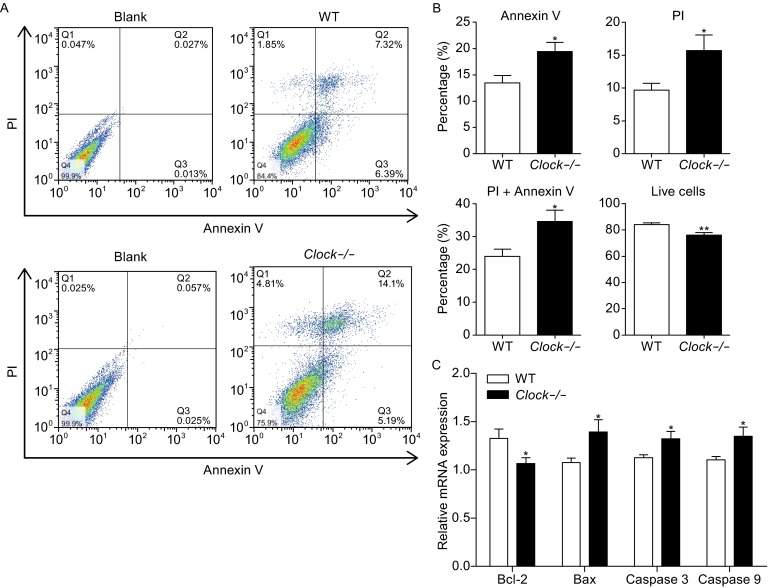
***Clock*** **silencing accelerated cell death in mESCs**. (A) Apoptosis and overall cell death of the wild type and *Clock*-/- mESCs were analyzed by flow cytometry using the Annexin V and PI assay, respectively. *Clock*-/- mESCs exhibited increased apoptosis and overall cell death. (B) Percentage of apoptotic (Annexin V) and dead (PI) cells calculated from six independent experiments. Data were expressed as means ± S.D. (C) Relative mRNA expression levels of apoptosis-related genes *Bax, Bcl-2,* cleaved *caspase-3*, and *caspase-9* in wild type and *Clock*-/- mESCs. The mRNA expression levels were normalized to that of the endogenous *GAPDH*. Data represent the average of three independent experiments and are expressed as means ± S.D. Statistical significance was calculated using the Student’s *t*-test: * denotes *P* < 0.05, ** denotes *P* < 0.01, *** denotes *P* < 0.001 compared with the Ctrl group, ns, not significant

The decreased proliferation of the *Clock* knockout mESCs suggested either a slowdown of cell cycle or increased cell death in mESCs. To test our hypotheses, we first examined the expression of cell cycle-related markers *C-Myc, CyclinD1, CDK1, CDK2, PCNA,* and *P27* by real-time PCR in the *Clock* knockout mESCs and compared with those in the wild type mESCs. We found that the expression levels of *C-Myc, CyclinD1, CDK1, CDK2,* and *PCNA*, which facilitated cell proliferation, were lower in *Clock* knockout mESCs than in the wild type mESCs (*P* < 0.05, *P* < 0.01). The P27 mRNA expression, which restrained cell proliferation, was higher in *Clock* knockout mESCs than in the wild type mESCs (Fig. 4B). These results indicated that *Clock* silencing in mESCs decreased the cell cycle. To determine whether the decreased proliferation also resulted from increased cell death, we further investigated cell death in the *Clock* knockout mESCs and the wild type mESCs. Based on six independent experiments using FACS analysis of apoptosis with Annexin V and PI (Propidium Iodide), respectively, indicated that the *Clock* knockout mESCs exhibited significantly higher rate of apoptosis and cell death compared with the wild type mESCs (*P* < 0.05 and *P* < 0.01) (Fig. 5A and B). To further verify this result, we determined the mRNA expression levels of apoptosis-related markers *Bax, Bcl-2, caspase 3,* and *caspase 9* in the *Clock* knockout mESCs and the wild type mESCs. As shown in Fig. 5C, the mRNA expression levels of *Bax, caspase 3,* and *caspase 9*, which were associated with enhanced apoptosis, were higher in the *Clock* knockout mESCs than in the wild type mESCs (*P* < 0.05). The expression level of *Bcl-2*, which was associated with decreased cell apoptosis, was lower than in the wild type mESCs (*P* < 0.05). These results demonstrated that silencing of Clock increased cellular apoptosis, resulting in increased cell death of mESCs.

### *Clock* is required for development of circadian oscillator during mESCs differentiation

**Figure 6:**
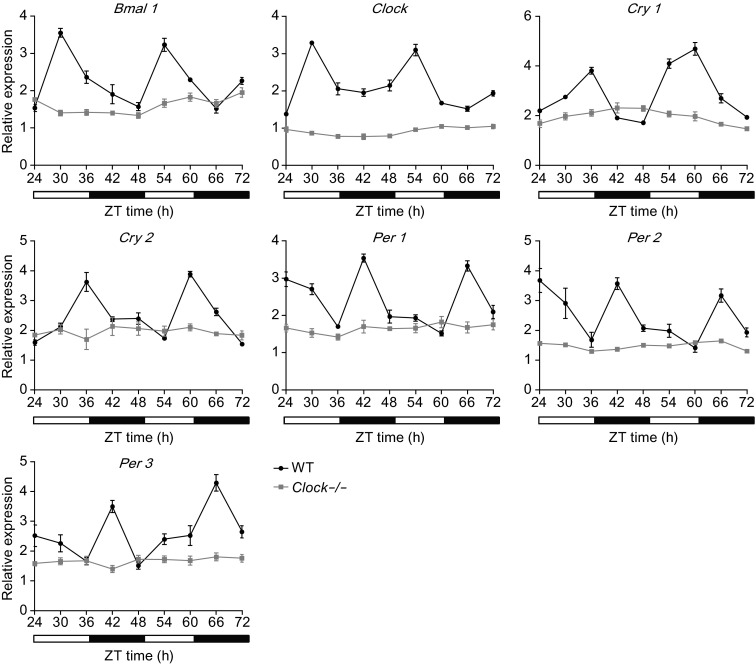
***Clock*** **silencing disturbed the circadian oscillations during mESC differentiation**. Relative mRNA expression of core clock genes *Bmal1, Clock, Cry1, Cry2, Per1, Per2*, and *Per3* in spontaneously differentiated wild type and *Clock*-/- mESCs after serum shock induced biological rhythms. ZT0 to 72 represent 8:00 am to 8:00 am of day 4, respectively. The mRNA expression levels were normalized to that of the endogenous *GAPDH*. Compared with wild type mESCs, *Clock*-/- mESCs showed no circadian oscillation in the expression of core clock genes. Data represent the average of three independent experiments and are presented as means ± S.D

Pluripotent mESCs do not exhibit molecular circadian oscillations. However, circadian rhythms developed during the differentiation of pluripotent mESCs into downstream lineages (Yagita et al., 2010). Whether silencing of the *Clock* gene altered the development of circadian oscillation of mESCs during differentiation remains unknown. Therefore, we analyzed the expression of the core clock genes *Bmal1, Clock, Per*, and *Cry* on day 15 after spontaneous differentiation of the *Clock* knockout mESCs and wild type mESCs. Consistent with a previous report, the wild type mESCs developed circadian oscillations on day 15 following spontaneous differentiation. In contrast, the *Clock* knockout mESCs exhibited no circadian oscillation on day 15 after spontaneous differentiation (Fig. 6). These results indicated that silencing of the gene *Clock* disrupted the development of circadian oscillations during the differentiation of pluripotent mESCs.

### *Clock* knockout in mESCs accelerated spontaneous differentiation

**Figure 7:**
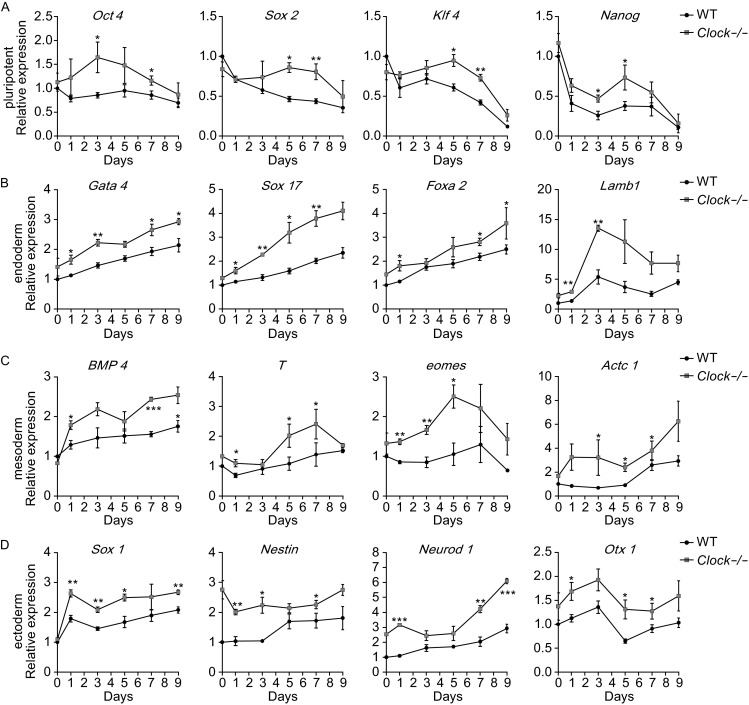
***Clock*** **silencing promoted the expression of genes controlling the three embryonic germ layers in spontaneously differentiated mESCs**. (A) Relative mRNA expression of pluripotent markers *Oct4, Sox2, Nanog,* and *Klf4* after spontaneous differentiation in wild type and *Clock*-/- mESCs. Expression of the pluripotent genes was gradually decreased in wild type and *Clock*-/- mESCs. (B) Relative mRNA expression of endoderm markers *Gata4, Sox17, Foxa2, and Lamb1* after spontaneous differentiation in wild type and *Clock*-/- mESCs. *Clock*-/- mESCs exhibited a higher expression of these endoderm genes. (C) Relative mRNA expression of mesoderm markers *BMP4, T, Eomes,* and *Actc1* after spontaneous differentiation in wild type and *Clock*-/- mESCs. *Clock*-/- mESCs exhibited a higher expression of mesoderm genes. (D) Relative mRNA expression of ectoderm markers *Nestin, Sox1, Neurod1,* and *Otx1* after spontaneous differentiation in wild type and *Clock*-/- mESCs. *Clock*-/- mESCs exhibited a higher expression of these ectoderm genes. The mRNA expression levels were normalized with that of the endogenous *GAPDH*. Data represent the average of three independent experiments and are presented as means ± S.D. Statistical significance was calculated using the Student’s *t*-test: *denotes *P* < 0.05, **denotes *P* < 0.01, ***denotes *P* < 0.001 compared with the Ctrl group, ns, not significant

**Figure 8:**
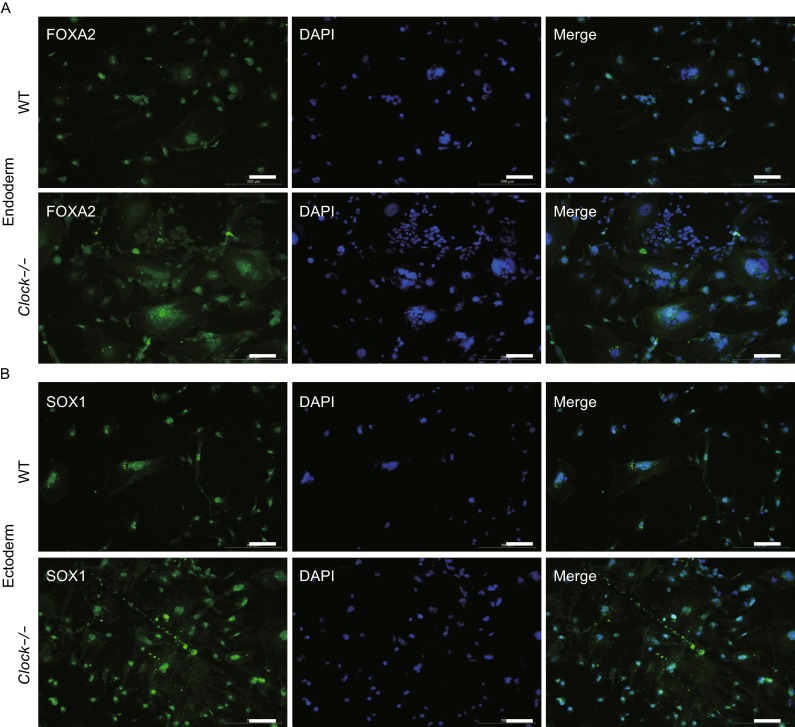
***Clock*** **-/- mESCs expressed higher endoderm marker FOXA2 and ectoderm marker SOX1 on day 9 after spontaneous differentiation**. (A and B) Immunofluorescence staining of the endoderm marker FOXA2 and the ectoderm marker SOX1 in spontaneously differentiated wild type and *Clock*-/- mESCs on day 9. *Clock*-/- mESCs exhibited relatively stronger expression of both FOXA2 and SOX1. Bars, 200 μm

We next investigated whether Clock knockout and disruption of the normal circadian oscillations affected mESCs differentiation. 1 × 10^4^ of wild type mESCs or *Clock* knockout mESCs were cultured in differentiation medium. Surprisingly, after day 7, *Clock* knockout mESCs showed more complex cellular structures as well as faster proliferation rate after spontaneous differentiation (Fig. S3A). The mRNA expression levels of the pluripotent markers *Oct4, Sox2, Nanog,* and *Klf4* were gradually decreased in both the *Clock* knockout mESCs and wild type mESCs after spontaneous differentiation (Fig. 7A), indicating an exit from the pluripotent state. Spontaneous differentiation also led to a gradual increase in the mRNA expression levels of the endoderm markers *Gata4, Sox17, Foxa2,* and *Lamb1* (Fig. 7B), the mesoderm markers *BMP4, T, Eomes,* and *Actc1* (Fig. 7C), as well as the ectoderm markers *Nestin, Sox1, Neurod1,* and *Otx1* (Fig. 7D). Interestingly, the mRNA expression levels of the above markers in the three embryonic germ layers (ectoderm, mesoderm, and endoderm) in the *Clock* knockout mESCs were relatively higher than in the wild type mESCs. The *Clock* knockout mESCs showed accelerated differentiation compared with wild type mESCs. To further verify these observations, we used immunofluorescence staining of the endoderm marker FOXA2 and the ectoderm marker SOX1 in both the *Clock* knockout mESCs and wild type mESCs on day 9 after spontaneous differentiation. The FOXA2 and SOX1 stained strongly in the *Clock* knockout mESCs than in the wild type mESCs (Fig. 8A and B). These results suggested that knockout the *Clock* triggered spontaneous differentiation of mESCs.

## DISCUSSION

In this study, we investigated the role of the core circadian gene *Clock* in the maintenance and differentiation of pluripotent stem cells. We used the CRISPR/CAS9 genome editing tool to induce frameshift mutations in exon 2 of the mouse *Clock* gene and completely ablated its protein expression. This is the first report of a complete knockout of *Clock* expression in pluripotent stem cells. Previous studies mostly used siRNA/shRNA-mediated silencing of *Clock* expression (Mukherjee et al., 2010; Tobback et al., 2012; Tracey et al., 2012; Liang et al., 2013; Li et al., 2015). The complete knockout of *Clock* in our study avoided any potential noise observed in partial knockdown studies involving siRNA/shRNA.

The knockout of the gene *Clock* did not disrupt the clonal morphology or pluripotency of mESCs. However, loss of *Clock* significantly decreased the proliferation and increased the cell death of mESCs. Our findings are consistent with other studies that reported a potential relationship between *Clock* gene and apoptosis as well as cell cycle regulation. One study demonstrated that upregulation of several pro-apoptic genes in the spleen of *Clock* mutant mouse (Gaddameedhi et al., 2015). Another study also reported that the *Clock* gene controlled the expression of key cell cycle-related regulators, such as *Cdc2, Wee1, P21, PCNA* and *Cdk2* in the intestine (Peyric et al., 2013). Our data also showed that the expression of cell cycle-related proteins in *Clock* knockout mESCs, was affected. The  expression of anti-apoptotic protein *Bcl-2* significantly decreased in *Clock* knockout mESCs. The expression of pro-apoptotic protein *Bax*, *cleaved caspase-3,* and *caspase-9* significantly increased in *Clock* knockout mESCs. These data suggested a disruption in the balance of mitochondria and activation of the caspase cascade in *Clock* knockout mESCs (Liang et al., 2013). *Clock* may, thus, contribute to the maintenance of normal proliferation by controlling the balance of cell cycle and apoptosis in mESCs. Overall, our results provide some new insights into the function of the *Clock* gene in the regulation of cell cycle and apoptosis of mESCs.

To consider the potential influence of off-target gene modifications (Yee, 2016), we detected the most probable ten off-target modification sites which were designed at the CRISPR website (http://crispr.mit.edu/) (Supplemental Table. 3) and found that there was no off-target gene modification (Supplemental Fig. 2), which excluded the potential influence of off-target gene modifications. And we further considered the potential influence of alternative cellular roles for *Clock* such as the transcription factor neuronal PAS domain protein 2 *NPAS2* (*MOP4*), which was able to functionally substitute for the loss of *Clock* in the SCN but not in peripheral tissues in mice to regulate circadian rhythmicity (DeBruyne et al., 2007a, b; Debruyne, 2008). In our study, we tested the mRNA expression level of *NPAS2* in the wild type mESCs and *Clock* knockout mESCs after spontaneous differentiation, and we found that both of them barely expressed *NPAS2* after spontaneous differentiation. This indicated that the nerve cells was very few in wild type mESCs and *Clock* knockout mESCs after spontaneous differentiation, so there wasn’t compensation of *NPAS2* for the loss of *Clock* (Fig. S3B). All in all, these data indicated that the phenotypes in these *Clock* knockout mESCs should mainly be due to the loss of *Clock* but not the influence of off-target gene modifications or loss of other *Clock* functions.

A recent study has shown that the mammalian circadian oscillator in ground state naïve pluripotent stem cells was not developed until after differentiation (Yagita et al., 2010). We also found in this study that naive mESCs did not exhibit circadian oscillations until differentiation, while *Clock* was required for the development of this circadian oscillator in mESCs after differentiation. This finding suggested that *Clock* mediated developmental functions in mammals. Indeed, we found that loss of *Clock* in mESCs triggered spontaneous differentiation. Several critical genes regulating development of the three emryonic germ layers were expressed higher in the *Clock* knockout mESCs, which exhibited accelerated spontaneous differentiation *in vitro*. The disruption of biological rhythms underlying differentiation of *Clock* knockout mESCs may drive these cells toward spontaneous differentiation upon withdrawal of pluripotent signals. Our study indicated that *Clock* medicated the differentiation of mouse pluripotent stem cells. Loss of *Clock* significantly decreased the proliferation and increased the cell death of mESCs indicated that *Clock* accurately regulated the development of mESCs. *Clock* knockout in mESCs accelerated spontaneous differentiation. Some studies have reported that *Clock* knockout in mice is associated with aging and chronic inflammation (Dubrovsky et al., 2010), indicating that this acceleration might be harmful. Our findings indicated that *Clock* knockout mESCs can conduct one tool to study these diseases. Yet, additional studies are required to uncover the roles of *Clock* in mammalian development.

In summary, we found that the core circadian gene *Clock* was dispensable for maintaining pluripotency in mouse pluripotent stem cells. However, it was required for maintaining regular proliferation and cell death in mESCs. And *Clock* was indispensable for the development of circadian oscillations after differentiation of pluripotent stem cells. Furthermore, our data indicated that *Clock* was essential for the differentiation of mouse pluripotent stem cells. Our findings may provide some new insights into the regulatory mechanisms of *Clock* in pluripotent stem cell development in mammals.

## MATERIALS AND METHODS

### Culture of mESCs

Mouse 129 ESCs were obtained from ATCC (American Type Culture Collection, Manassas, VA). The wild type and *Clock* knockout mESCs were cultured in the maintenance medium of Dulbecco’s Modified Eagle’s Media (DMEM, Gibco, USA) supplemented with 3.7 g/L sodium bicarbonate, 1% penicillin and streptomycin, 1.7 mmol/L L-glutamine, non-essential amino acid, 55 mmol/L beta mercaptoethanol, 5 ng/mL mouse leukemia inhibitory factor (LIF), 3 μmol/L EsK3β inhibitor, 1 μmol/L MEK inhibitor and 10% FBS. The mESC maintenance medium was used for spontaneous differentiation without supplementing LIF, the EsK3β inhibitor or the MEK inhibitor.

### Construction of the *Clock* knockout mESC line

To construct the *Clock* knockout mESC line, we utilized the clustered, regularly interspaced, short palindromic repeats (CRISPR)/CRISPR-associated protein 9 (CAS9) (CRISPR/CAS9) genomic editing tool. The CRISPR/CAS9 system is an efficient tool for genome engineering. It induces double-strand breaks (DSBs) and repair using the non-homologous end-joining (NHEJ) mechanism at any specific site. Briefly, one pair of independent oligo primers targeting exon 2 of the *Clock* gene was subcloned into the pX330 vector (Mizuno et al., 2014) (Addgene) to obtain the pX330-Clock plasmid. The primer sequences generating the guide RNA (gRNA) targeting the *Clock* gene were TCCATCTTTCTCGCGTT, then the complementary sequences were AGGTAGAAAGAGCGCAA. As the transcriptional promoter of PX330 plasmid was U6, we replaced base T to base G for the first base was G to be effective transcription. With enzyme sites, the insertion sequences were as follows: oligo1: 5′-CACCGCCATCTTTCTCGCGTTACC-3′, oligo2: 5′-AAACGGTAACGCGAGAAAGAT GGC-3′. The pX330-Clock plasmid was transfected into single mESCs by electroporation. The correct knockout of *Clock* gene expression was confirmed by Western blot analysis and gene sequencing.

To consider the potential influence of off-target gene modifications (Yee, 2016), we detected the most probable ten off-target modification sites which were designed at the CRISPR website (http://crispr.mit.edu/), whose off-target cleavage site sequences and gene numbers were as follows in Table S3. Specific PCR primers were designed according to the target gene sequences of mouse (Table S3).

### Quantitative real-time PCR

The mRNA expression levels of *GAPDH, Oct4, Sox2, Klf4, Nanog, Zfp296, Eras, Dax1, Esg1, C-Myc, PCNA, CDK1, CDK2, CyclinD1, P27, Gata4, Sox17, Foxa2, Lamb1, BMP4, T, Eomes, Actc1, Nestin, Sox1, Neurod1, Otx1, Bcl-2, Bax, cleaved caspase-3,* and *caspase-9* were quantified by RT-PCR. Specific PCR primers were designed according to the target gene sequences of mouse (Supplemental Table. 1). Cells were washed by ice-cold Phosphate buffer solution (PBS, NaCl 137 mmol/L,KCl 2 mmol/L, Na_2_HPO_4_ 10 mmol/L, KH_2_PO_4_ 10 mmol/L pH 7.4). Total RNA was extracted by using Trizol reagent (Invitrogen, USA). Qualities of extracted RNA qualities were measured by OD_260_/OD_280_ ratios which ranged from 1.9 to 2.1. The cDNA was obtained through reverse transcription kit according to manufacturer’s instructions (TOYOBO, Japan). The amplification mixture comprised 1 μL of RT reaction mix, 10 μL of SYBR® Premix Ex Taq TM (2×) (TaKaRa, China), 0.5 μL of 10 μmol/L each of primers and 8.5 μL ddH_2_O. Reactions were performed on a fluorescence temperature cycler (Bio-Rad, Hercules, CA, USA). The PCR conditions for *Clock* and *GAPDH* were as follows: one cycle of 3 min at 95°C; 35 cycles of 15 s at 95°C, 30 s at 58°C, 30 s at 72°C. The primer sequences are listed in Supplemental Table 1. The threshold cycle (CT) in RT-PCR was analyzed using the 2^-ΔΔCt^ method

### Western blot

Total protein was extracted from the treated cells using Radio-Immunoprecipitation Assay (RIPA) lysis buffer (50 mmol/L Tris/HCl pH 7.4, 150 mmol/L NaCl, 1% Nonidet-P40, 0.5% Sodium deoxycholate, 0.1% SDS). The extraction and isolation of nuclear protein were performed according to the instructions in the Nuclear and Cytoplasmic Protein Extraction Kit (Beyotime, China). Equal amounts (30–50 μg) of protein extracted from cells were boiled at 100°C in 1× SDS loading buffer for 10 min and then were loaded and separated by sodium dodecyl-sulfate (SDS)-polyacrylamide gel electrophoresis (PAGE). Proteins were transferred to a polyvinylidene fluoride (PVDF) membrane (Millipore, Billerica, MA, USA), blocked by 5% nonfat dry milk in Tris-buffered saline containing Tween-20. The membranes were incubated with antibodies against *PKM2* (Cell Signaling), *CK19* (Abcam), and *EpCAM* (Abcam). Signals were detected with horseradish peroxidase (HRP)-conjugated goat anti-mouse or goat anti-rabbit IgG. Immunoreactive bands were visualized by enhanced chemiluminescence (ECL, Amersham Biosciences), which was developed and quantified using an imaging system (Tanon, China).

### Alkaline phosphatase staining

The sections were washed with cold 1 × PBS three times, treated with 4% paraform- aldehyde for 1 to 2 min, washed with cold 1 × PBS twice, and with 1 × Tris buffer solution tween (TBST, 50 mmol/L Tris, 150 mmol/L NaCl, 1% Tween-20) once, followed by staining with Alkaline Phosphatase Kit (Millipore, USA) according to the manufacturer’s instructions. Images were taken by a Leica DMi8 microscope (Leica, Germany) and analyzed using Image Pro Plus 6.0 software (Media Cybernetics, Rockville, MD).

### Teratoma formation assay

To study the pluripotency of wild type mESCs and *Clock* knockout mESCs *in vivo*, we performed teratoma formation assay which could conduct as a tool for monitoring pluripotency in stem cell research (Nelakanti et al., 2015). We injected 1 × 10^7^ wild type mESCs and *Clock* knockout mESCs into the oxters of immunodeficiency mice respectively. 1–2 cm teratoma formed after four weeks. Six weeks later, we detected the differential capacity of wild type mESCs and *Clock* knockout mESCs by hematoxylin-eosin staining (HE staining). We found that both wild type mESCs and *Clock* knockout mESCs could differentiate to endoderm, mesoderm, and ectoderm layers cells, which indicated that they have similar potential of multilineage differentiation *in vivo*.

### Immunofluorescent staining

The sections were washed with cold 1 × PBS three times, and treated with 4% paraformaldehyde for 15 min. They were again washed with cold 1 × PBS three times, and treated with 5% Triton X-100 in Tris buffer solution (TBS, 50 mmol/L Tris/HCl pH 7.4, 150 mmol/L NaCl) for 20 min at room temperature (RT) (membrane proteins do not need this step). After another wash with cold 1 × PBS three times for 5 min, they were treated with normal goat serum for 30 min at RT, followed by the addition of primary antibodies (1:200) and incubation in a wet box for 2 h at RT or 4 °C overnight. Fluorescent secondary antibodies (1:350) were added and the cells were incubated for 30 min or 1 h at RT, washed three times for 5 min each with PBST (1% Tween in PBS buffer). It was followed by staining with 2-(4-Amidinophenyl)-6-indolecarbamidine dihydrochloride (DAPI) (1:1000) for 5 min at RT, and washed three times for 5 min each with PBS. After treatment with a fluorescence quenching agent, the specimens were sealed and photographed under fluorescence and laser confocal microscope. The images were analyzed using Image Pro Plus 6.0 software (Media Cybernetics, Rockville, MD).

### Cell proliferation curve

Cell viability and number was detected using a cell counting chamber. Wild type mESCs and *Clock* knockout mESCs were transferred to 12-well and 6-well plates (Corning Inc., Corning, New York, USA) at 1 × 10^4^ cells/well and cultured at 6 different time points (24, 48, 72, 96, 120 and 144 h). At the indicated time, cells were digested with 0.05% trypsin, and 20 μL of the resuspended cells were collected in the cell counting chamber (Nexcelom) and counted using the Cellometer Mini software.

### Induction of biological rhythms with horse serum shock

Briefly, we induced the biological rhythms of wild type mESCs and *Clock* knockout mESCs after spontaneous differentiation with horse serum shock as previously described (Balsalobre et al., 1998; Xiang et al., 2012). The wild type mESCs and *Clock* knockout mESCs were cultured in the mESC maintenance medium and differentiated spontaneously for 15 days in the differentiation medium. The medium was removed and cells were washed with 1 × PBS, followed by addition of the differentiation medium without FBS for 24 h. The medium was removed and cells were washed with 1 × PBS. A new differentiation medium containing 50% horse serum was added to the cells. After incubation for 2 h, the medium was removed and washed with 1 × PBS, and replaced with the original differentiation medium.

### Annexin V and PI assay

The FITC Annexin V and Propidium Iodide (PI) Apoptosis Detection Kit II (Pharmingen, USA) were used to analyze apoptosis and cell death rate using flow cytometry. The wild type mESCs and *Clock* knockout mESCs were collected and washed twice with cold PBS. The resuspended cells in 100 μL of 1 × binding buffer were transferred to a 1.5 mL culture tube, and 5 μL of FITC Annexin V and PI were added to each tube. After gentle vortexing, the cells were incubated for 15 min at room temperature in the dark. Finally, 400 μL of the 1 × binding buffer was added to each tube, and analyzed by flow cytometry in 1 h.

### Statistical analysis

All the data were expressed as means ± SD. The statistical significance of differences between the experimental groups was determined using one-way ANOVA by Student’s t-test (SPSS 11.0, SPSS Inc., Cary, NC). Probability values (*p*) of less than 0.05 were considered statistically significant.

## Electronic supplementary material

Below is the link to the electronic supplementary material.

Supplementary material 1 (PDF 392 kb)

Supplementary material 2 (PDF 220 kb)
