# Supplementary material for: Role of circadian gene Clock during differentiation of mouse pluripotent stem cells
Source: Protein Cell. 2016 Sep 23;7(11):820–32. doi: 10.1007/s13238-016-0319-9 (PMC5084156; doi:10.1007/s13238-016-0319-9)
Supplement: Supplementary file 1 — Supplementary material 1 (PDF 392 kb) [file 13238_2016_319_MOESM1_ESM.pdf]

Supplemental Figure 1

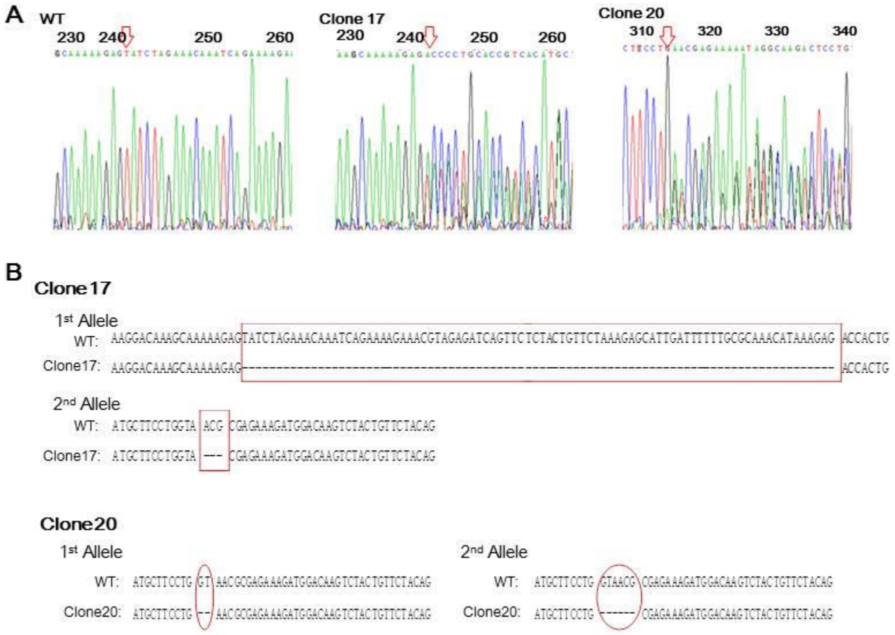

Supplemental Figure 1

(A) The positive homozygous mESC clone 17 and clone 20 showed modified gene sequence on exon 2 in both alleles of the Clock gene. (B) Sequencing results of the TA cloning of PCR products flanking the CRISPR/CAS9 cutting site for clone 17 and clone 20.

Supplemental Figure 2

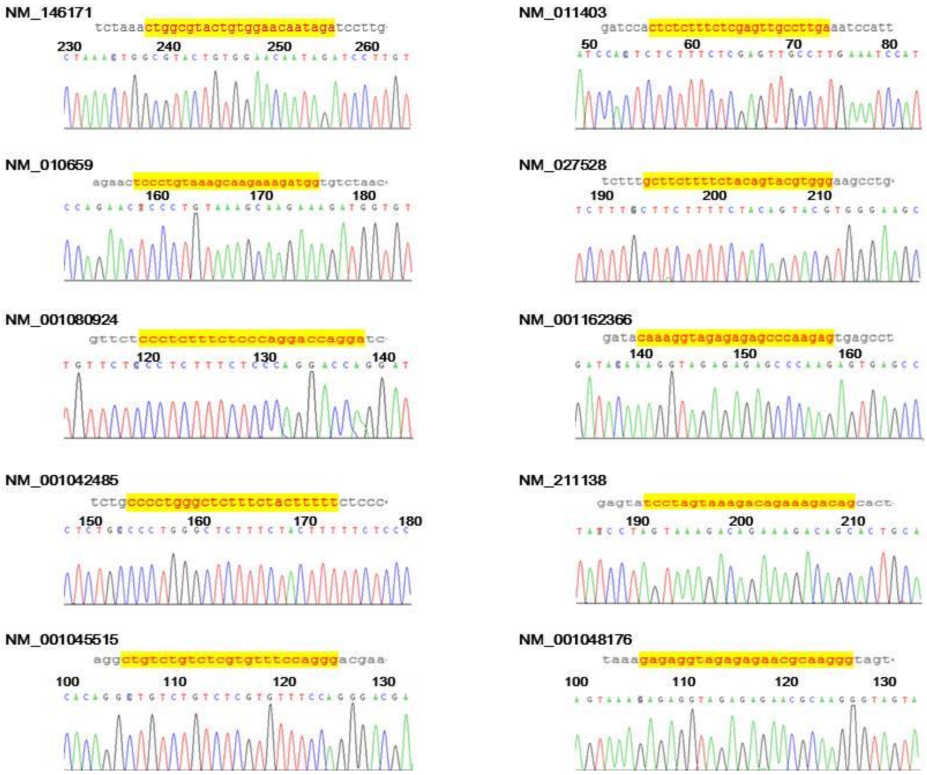

Supplemental Figure 2

The yellow shaded parts were the most probable ten off-target modification sites. The corresponding genes were NM_146171, NM_011403, NM_010659, NM_027528, NM_001080924, NM_001162366, NM_001042485, NM_211138, NM_001045515 and NM_001048176. Specific PCR primers were designed according to the target gene sequences of mouse (Supplemental Table. 3). The Sanger's sequencing indicated that there was no off-target gene modification at these most probable ten off-target modification sites.

Supplemental Figure 3

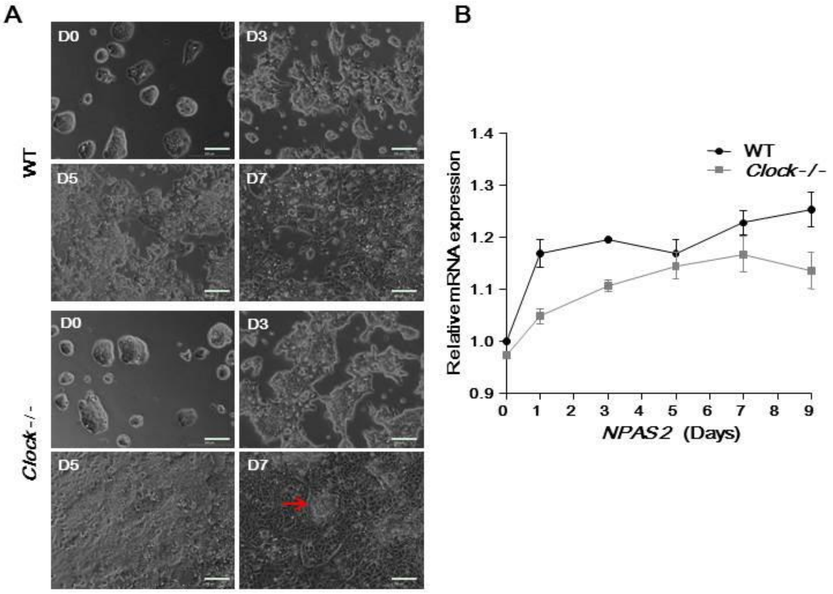

Supplemental Figure 3

(A) Phase images of wild type and Clock-/- mESCs on days 0, 3, 5, and 7 after spontaneous differentiation. The red arrows indicate complex cellular structures found in Clock-/- mESCs on day 7 after spontaneous differentiation. Bars, 200 m. (B) Relative mRNA expression level of NPAS2 after spontaneous differentiation in wild type and Clock-/- mESCs. The mRNA expression levels were normalized with that of the endogenous GAPDH. Data represent the average of three independent experiments and are presented as means ± S.D.
