# Supplementary material for: Role of circadian gene Clock during differentiation of mouse pluripotent stem cells
Source: Protein Cell. 2016 Sep 23;7(11):820–32. doi: 10.1007/s13238-016-0319-9 (PMC5084156; doi:10.1007/s13238-016-0319-9)
Supplement: Supplementary file 2 — Supplementary material 2 (PDF 220 kb) [file 13238_2016_319_MOESM2_ESM.pdf]

Supplemental Table 1. The primers used for qPCR in this study.

Gene name	Primer (5'-3')
GAPDH	Forward :AACGACCCCTTCATTGACCT<br>Reverse: TGGAAGATGGTGATGGGCTT
Oct4	Forward : CCTTGCAGCTCAGCCTTAAG<br>Reverse: GCGATGTGATGATCTGCTG
Sox2	Forward :CCAAGATGCACAACTCGGAT<br>Reverse: CTCCGGGAAGCGTGTACTTA
Klf4	Forward :AGAAGTGTGACAGGGCCTTT<br>Reverse: TCGTGGGAAGACAGTGTGAA
Nanog	Forward :CGCCATCACACTGACATGAG<br>Reverse: AGAAGAATCAGGGCTGCCTT
Eras	Forward :CTACTGGAAGGAAGTGGCCA<br>Reverse: TATCTGCTGCAACTGGTCCA
Dax1	Forward :CTATGTGTGCGGTGAAGAGC<br>Reverse: TGGAAGCAGGGCAAGTACTT
Zfp296	Forward :GTCAACTCCAAACGTCCTCG<br>Reverse: TGGATTCTGAGATGGGGTCG
Esg1	Forward :GGCAACTGTTATGGCGTGAA<br>Reverse: TCCTGACGAAGCTGTGTCAT
C-Myc	Forward :CACACAACGTCTTGGAACGT<br>Reverse: CGTCTGCTTGAATGGACAGG

PCNA	Forward :TATGCCGAGACCTTAGCCAC<br>Reverse: GGTTACCGCCTCCTCTTCTT
CDK1	Forward :TCCTGGGCAGTTCATGGATT<br>Reverse: TCCTGGGCAGTTCATGGATT
CDK2	Forward :AATGCAGAGGGGTCCATCAA<br>Reverse: CTCCAGATATCCACGGCTGT
CyclinD1	Forward :AGAAGTGCGAAGAGGAGGTC<br>Reverse: CTTAGAGGCCACGAACATGC
P27	Forward :GACCAAATGCCTGACTCGTC<br>Reverse: TCTTCTGTTCTGTTGGCCCT
Gata4	Forward :AAAACGGAAGCCCAAGAACC<br>Reverse: ATAGTGAGATGACAGCCCGG
Sox17	Forward :GCCCTTTGTGTATAAGCCCG<br>Reverse: GCAATAGTAGACCGCTGAGC
Foxa2	Forward :CTACACACACGCCAAACCTC<br>Reverse: TTGAAGGAGAGAGAGTGGCG
Lamb1	Forward :AGCTTGCGTGTGTTTGTGAT<br>Reverse: CAATGTTGTGGTGGCACTGA
BMP4	Forward :CTTCAACCTCAGCAGCATCC<br>Reverse: GATGAGGTGTCCAGGAACCA
T	Forward :ACCCAGCTCTAAGGAACCAC<br>Reverse: GCTGGCGTTATGACTCACAG

Eomes	Forward :TCGTGGAAGTGACAGAGGAC<br><br>Reverse: TAGTTGTCCCGGAAGCCTTT
Actc1	Forward :TGCCGATCGTATGCAAAAGG<br><br>Reverse: GGCCTGCCTCATCATACTCT
Nestin	Forward :AGGTGTCAAGGTCCAGGATG<br><br>Reverse: AAGGAAGCAGACTCAGACCC
Neurod1	Forward :AGGAGGAGGATCAAAAGCCC<br><br>Reverse: GGGTCTTGGAGTAGCAAGGT
Sox1	Forward :ACACAGTGGGGATAACAGCA<br><br>Reverse: GCCACGCCCTCATGATATTG
Otx1	Forward :CGGAAGCTATGGTCAGGGAT<br><br>Reverse: TGAAGATTGGCTCAGTGGGT
Bcl-2	Forward :AACTCTTCAGGGATGGGGTG<br><br>Reverse: TACTCAGTCATCCACAGGGC
Bax	Forward :GAGACACCTGAGCTGACCTT<br><br>Reverse: GTCCACGTCAGCAATCATCC
cleaved caspase-3	Forward :CAGCCAACCTCAGAGAGACA<br><br>Reverse: ACAGGCCCATTTGTCCCATA
cleaved caspase-9	Forward: CCAAGATGCACAACTCGGAG<br><br>Reverse: ATCCGGGTGTTCCTTCATGT
Bmal1	Forward: AGGCCCACAGTCAGATTGAA<br><br>Reverse: GAACAGCCATCCTTAGCACG

Clock	Forward: ATGCCACAGAACAGTACCCA<br>Reverse: TTGTGTGGCGAAGGTAGGAT
Cry1	Forward: GGCAACTGTTATGGCGTGAA<br>Reverse: TCCTGACGAAGCTGTGTCAT
Cry2	Forward: TTCTACACGGCAGCTACCAA<br>Reverse: TGCCTCAGTTGGGTCATGAT
Per1	Forward: CACCCTGATGACCCACTCTT<br>Reverse: CCTCCTCCTCCATAGCCAAG
Per2	Forward: AGCGTTACCTCTGAGCACAT<br>Reverse: ATGGATGCAACCTGGTCAGA
Per3	Forward: CCCTGTCTGTCCTCTGTTGT<br>Reverse: CTCTCTCCTTTGGCTGGTGA

Supplemental Table2. The clones used for detecting mutations in this study.

Total clones (number)	31
The sequences of bases have changed in clones	8 (25.8%)
CLOCK protein expression has changed in clones	6 (19.4%)
Homozygous clones	3 (9.68%)
Heterozygous clones	3 (9.68%)

We totally picked out 31 single mESC clones, and 8 (25.8%) of them (clone 5, clone 13,

clone 17, clone 19, clone 20, clone 23, clone 24, and clone 28) showed sequence modifications at the CRISPR/CAS9 cutting site. CLOCK protein expression has changed in 6 clones (19.4%) (clone 17, clone 19, clone 20, clone 23, clone 24, and clone 28) of the 8 positive clones with Western blotting. The results indicated that CLOCK protein expression was totally ablated in three clones (9.68%) (clone 17, clone 19, and clone 20), for homozygous clones; CLOCK protein expression was not totally ablated in three clones (9.68%) (clone 23, clone 24, and clone 28), for heterozygous clones.

Supplemental Table 3. The most probable ten off-target modification sites and the primers used for detecting off-target effects in this study.

Position	Sequence	Score	Ontarget	Gene	Primer
76694865	TCCATCTTTCTC GCGTTACCAGG	100	True	NM_007715	
125121193	TCTATTGTTCCA CAGTACGCCAG	0.751669231	False	NM_146171	Fwd: TCTGGCTATCCGCTTCCCTAA
					Rev: CGCTGTCTTCCGCACTTGTT
102221420	TCAAGGCAACTC GAGAAAGAGAG	0.526289801	False	NM_011403	Fwd: CTTCCCACAGAGCAAACAGC
					Rev: CCAGCCAAGCCTGAAAGC
99910981	TCCCTGTAAAGC AAGAAAGATGG	0.509948268	False	NM_010659	Fwd: AGGACCAAGTGAGTTATTCAGCAT
					Rev: GTTCCTCTTGAGACACAGCAGC
172444809	GCTTCTTTTCTA CAGTACGTGGG	0.485473295	False	NM_027528	Fwd: GAGCAAGATGAAAATGGAAGCA
					Rev: AAAGCCATCCATTCTGCCTCT

5179756	TCCTGGTCCTGG GAGAAAGAGGG	0.36666 4223	False	NM_0010809 24	Fwd: TTATCACACAGAGCCAAATCACC<br>Rev: GGTTGAGGAGGGCTCTCTTTAT
66796510	CAAAGGTAGAGA GAGCCCAAGAG	0.33961 9976	False	NM_0011623 66	Fwd: TAGAGAGATGGTGGCGTTTGTTT<br>Rev: TGACCCTCGCTGCTATCCC
13625204 5	AAAAAGTAGAAA GAGCCCAGGGG	0.25987 7595	False	NM_0010424 85	Fwd: GGCTGCTGCTGGCTTCCTA<br>Rev: CAGCCATAGGAAAGACAATCACC
90993123	TCCTAGTAAAGA CAGAAAGACAG	0.24692 7836	False	NM_211138	Fwd: GACATTTTGGGTAAAGGGAAGG<br>Rev: TTCAGGTGAGCAGGGAGGTAGT
90964219	CCCTGGAAACAC GAGACAGACAG	0.23525 4	False	NM_0010455 15	Fwd: TTAGAGCAGTCCTTAGAGACACCA<br>Rev: CCCAGTTTTGTTGTCTTATGAGC
79182745	GAGAGGTAGAGA GAACGCAAGGG	0.22815 1896	False	NM_0010481 76	Fwd: GACATCAGCATCAGAACCCTAAT<br>Rev: CACTGACCGTATGTGACCTGTAA
